# Cytidine deaminase activity increases in the blood of breast cancer patients

**DOI:** 10.1038/s41598-022-18462-8

**Published:** 2022-08-18

**Authors:** Géraldine Buhagiar-Labarchède, Rosine Onclercq-Delic, Sophie Vacher, Frédérique Berger, Ivan Bièche, Dominique Stoppa-Lyonnet, Mounira Amor-Guéret

**Affiliations:** 1 Institut Curie, PSL Research University, UMR 3348, 91405 Orsay, France; 2 CNRS UMR 3348, Centre Universitaire, Bat 110, 91405 Orsay, France; 3 Université Paris-Saclay, Centre Universitaire, Bat 110, UMR 3348, 91405 Orsay, France; 4 Department of Genetics, Institut Curie, Paris, France; 5 Department of Biostatistics, Institut Curie, Paris, France; 6 Université Paris Cité, Paris, France; 7 INSERM U830, Institut Curie, Paris, France

**Keywords:** Cancer, Genetics

## Abstract

Cytidine deaminase (CDA), an enzyme of the pyrimidine salvage pathway, deaminates cytidine, deoxycytidine and analogs, such as gemcitabine. Constitutive low levels of CDA activity have been reported in the blood of patients with hematological malignancies or suffering from gemcitabine toxicity. We previously reported that cellular CDA deficiency leads to genetic instability. We therefore hypothesized that constitutive CDA deficiency might confer a predisposition to cancer. We analyzed CDA activity and expression in blood samples from breast cancer (BC) patients with a suspected predisposition to the disease, and in healthy controls. Contrary to our hypothesis, we found that both CDA activity and mRNA levels were higher in blood samples from BC patients than in those from controls, and that this difference was not due to excess neutrophils. CDA activity levels were significantly higher in the serum samples of BC patients treated by radiotherapy (RT) than in those of untreated healthy controls, and hormone therapy in RT-treated BC patients was associated with significantly lower levels of CDA activity. A preliminary analysis of CDA activity in the serum of the very few BC patients who had undergone no treatment other than surgery suggested that the increase in CDA activity might be due to the breast cancer itself. Our findings raise important questions, which should lead to studies to elucidate the origin and significance of the increase in CDA activity in the serum of BC patients, and the impact of hormone therapy.

## Introduction

Cytidine deaminase (CDA) is an enzyme of the pyrimidine salvage pathway catalyzing the hydrolytic deamination of cytidine and deoxycytidine (dC) to uridine and deoxyuridine, respectively^1^. CDA also deaminates and inactivates nucleoside analogs, such as gemcitabine and cytosine arabinoside C, which are widely used to treat cancer^2^. Many studies have, therefore investigated the role of CDA in resistance to chemotherapies based on cytidine analogs^3^. CDA overexpression is a known marker of resistance to such chemotherapies, whereas low levels of CDA activity in the blood have been reported to lead to early severe toxicity, which affects 7–12% of cancer patients treated with gemcitabine^4^.

CDA enzyme activity naturally varies widely within populations and across tissues^5^, but the potential links between these variations and cancer have been little studied. We have shown that the pyrimidine pool disequilibrium resulting from CDA deficiency contributes to the typical genetic instability of cells from patients with Bloom syndrome (BS), a human autosomal recessive disease displaying one of the strongest known correlations between chromosomal instability and a high risk of cancer^6^. We also reported that CDA downregulation itself leads to an intracellular accumulation of dC and dCTP and reproduces several aspects of the genetic instability associated with BS, such as an increase in sister chromatid exchange (SCE) and ultrafine anaphase bridge (UFB) frequencies^6–10^. Moreover, CDA activity levels are frequently low in patients with hematological malignancies, suggesting that this “poor metabolizer” phenotype may constitute a risk for blood cancers^11^. Our results, showing that CDA deficiency leads to genetic instability, provide the basis of an explanation for these findings^11^. Thus, our finding that CDA deficiency leads to genetic instability, and those of studies reporting constitutively low levels of CDA activity in blood^4,11^, led us to hypothesize that constitutive CDA deficiency might confer a predisposition to cancer development.

We analyzed CDA mRNA and protein activity levels in blood samples from BC patients with a suspected predisposition to breast cancer (BC), and from healthy volunteers as controls, to determine whether a constitutive CDA deficiency might underlie some breast cancers.

## Results

### CDA mRNA and protein activity levels are significantly higher in serum samples from BC patients than in those from healthy donors

**Figure 1:**
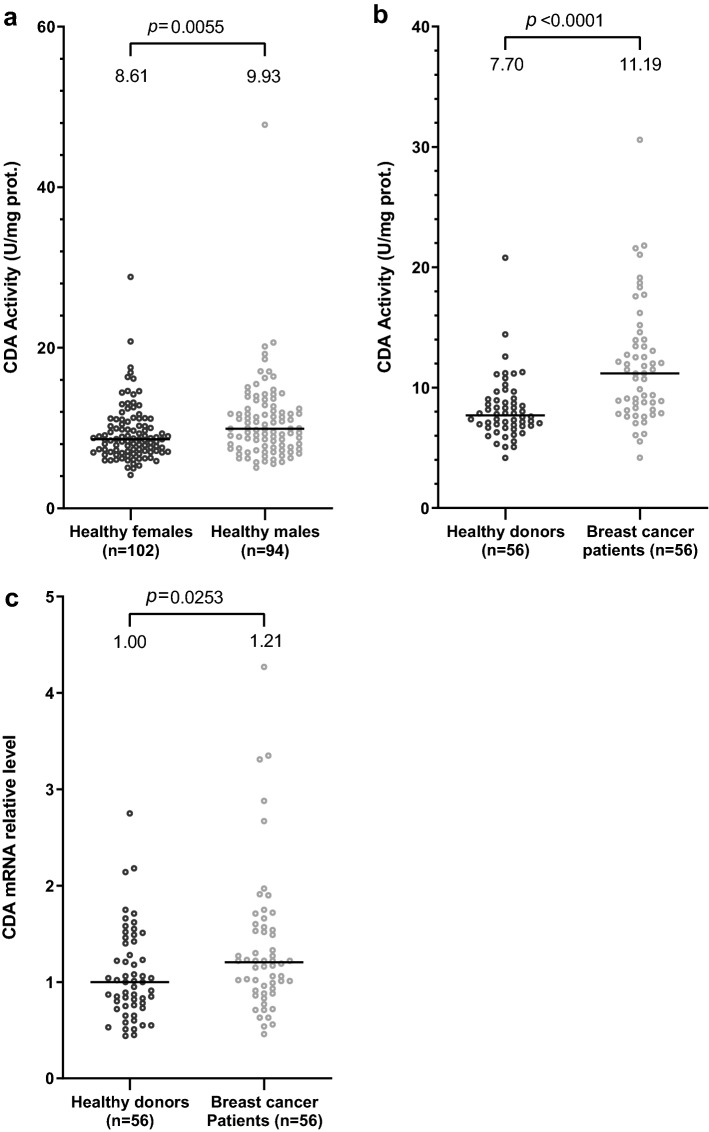
CDA activity and mRNA levels are significantly higher in the serum of BC patients than in that of healthy donors. (**a**) Scatter plot representation of CDA activity in enzymatic units/mg of protein for healthy female (in black) and healthy male (in gray) subjects. (**b**) Scatter plot representation of CDA activity in enzymatic units/mg of protein in matched healthy donors (in black) and BC patients (in gray). (**c**) Scatter plot representation of relative levels of CDA mRNA in matched healthy donors (in black) and BC patients (in gray). In (**b**) and (**c**), patients and controls were matched for age (± 4 years) and sex (5 male and 51 female subjects). For each panel, medians are represented by black horizontal lines and are indicated above each distribution. *n* is the number of individuals. Distributions were compared in Mann–Whitney tests or Wilcoxon matched-pairs signed-rank tests (see “Materials and methods” section). Values of *p* < 0.05 were considered statistically significant.

We collected blood samples from BC patients (*n* = 183) and from healthy donors as controls (*n* = 196) for comparisons of levels of CDA mRNA and protein activity. CDA activity levels have been reported to be significantly higher in the plasma of male than of female subjects^12^. The control population of healthy donors included far more male individuals (94 individuals) than the BC patient population (10 individuals), and recommendations indicate that CDA activity should be measured in serum rather than plasma^13^. We therefore first compared CDA activity between serum samples from female (*n* = 102) and male (*n* = 94) subjects from the control population of healthy donors. We found that CDA activity was slightly, but significantly (*p* = 0.0055), higher in serum samples from male subjects (median: 9.93 U/mg protein) than in serum samples from female subjects (median: 8.61 U/mg protein) (Fig. 1a). We therefore established homogeneous conditions for comparing patients and controls, by using R software to match patients and controls for age (± 4 years) and sex (5 male and 51 female subjects), with selection from the individuals (165 BC patients and 132 healthy donors) for whom we had both serum and mRNA samples (it was not possible to recover RNA from several blood samples for technical reasons). We found that both CDA activity and mRNA levels were significantly higher in the samples of BC patients than in the samples of controls (median: 11.19 vs. 7.70 U/mg protein for CDA activity, and 1.21 vs. 1.00 for CDA mRNA levels, respectively) (Fig. 1b,c). Careful analysis of the distribution of CDA activity and CDA mRNA levels in the matched populations of healthy donors and BC patients confirmed that the lowest values of CDA activity or mRNA levels were found in healthy donors and that, conversely, the highest values were found in BC patients (Supplementary Fig. 1a, b).

Serum CDA activity is considered to result from either leakage from damaged neutrophils^14^, or release during neutrophil breakdown, because neutrophils strongly express this enzyme and have a short half-life of about 6 to 8 h^15^. An analysis of blood count data from BC patients, conducted at the time of blood sample collection, showed no correlation between the higher levels of CDA activity in serum samples and higher neutrophil counts (Supplementary Fig. 2).

These results revealed that the constitutive CDA activity and mRNA expression were not lower in patients than in healthy donors. CDA deficiency is, therefore, not associated with a predisposition to the development of breast cancer in this cohort of patients, invalidating our leading hypothesis. Conversely, we found that CDA activity and mRNA levels were significantly higher in serum samples from BC patients than in those from healthy donors, and that this increase was not due to an increase in the number of neutrophils.

### CDA mRNA and protein activity levels in serum samples from BC patients treated by radiotherapy are higher than those in untreated healthy donors

We then addressed the question as to whether the increase in CDA activity and mRNA levels in BC patients was due to the disease or cancer treatment.

**Figure 2:**
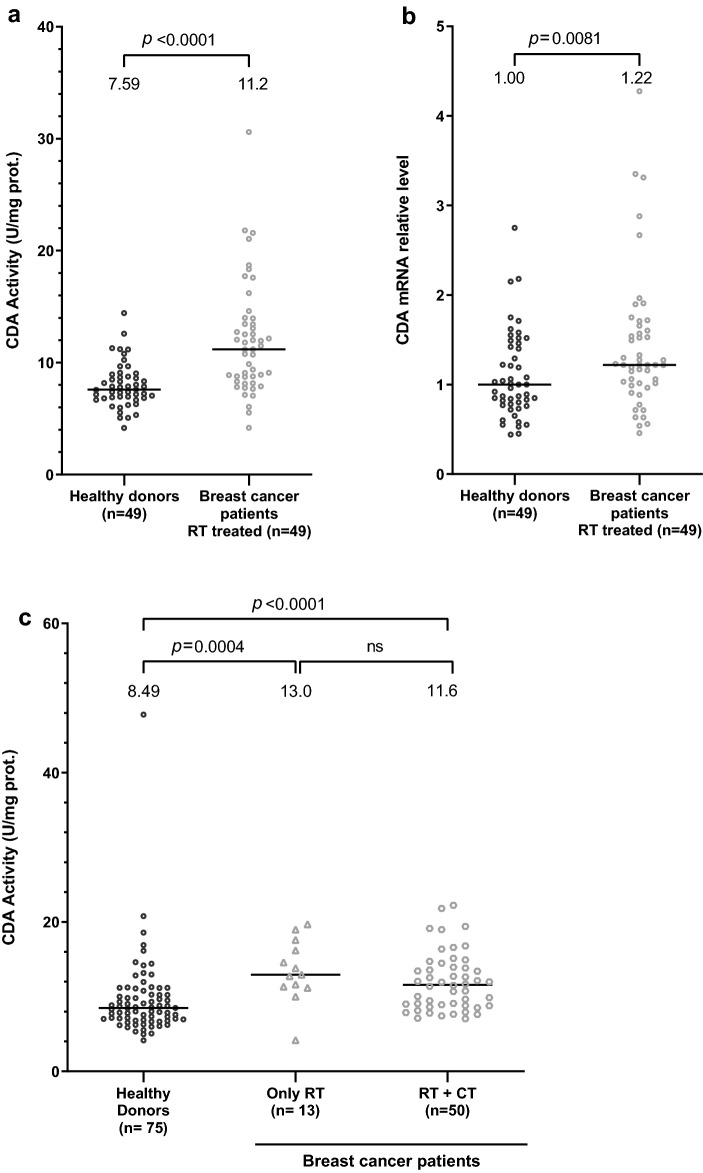
CDA activity and mRNA levels are significantly higher in serum samples from RT-treated BC patients than in those from untreated healthy donors. (**a**) Scatter plot representation of CDA activity in enzymatic units/mg of protein in matched healthy donors (in black) and RT-treated BC patients (in gray). (**b**) Scatter plot representation of relative levels of CDA mRNA in matched healthy donors (in black) and RT-treated BC patients (in gray). (**c**) Scatter plot representation of CDA activity in enzymatic units/mg of protein in healthy donors (in black) and BC patients treated with RT (with or without HT; triangle in gray), or treated with RT plus CT, (with or without HT; circle in gray) (one patient for whom we did not have CT treatment information was not included in this analysis). For each panel, medians are represented by black horizontal lines and are indicated above each distribution. *n* is the number of individuals. Distributions were compared in Kruskal–Wallis tests or in Wilcoxon matched-pairs signed-rank tests (see “Materials and methods” section). Values of *p* < 0.05 were considered statistically significant.

Only 10 of the 183 BC patients had received no treatment other than surgery, making it impossible to compare untreated patients with patients treated by radiotherapy (RT), chemotherapy (CT) and/or hormone therapy (HT). Nevertheless, as most of the patients were treated by RT (162) (Table 1), including 49 of the 56 matched BC patients (Supplementary Table 1), we compared RT-treated patients with matched untreated healthy donors, even though this analysis would not allow us to discriminate between the potential effects of RT and of the cancer itself, and confirmed that both CDA activity and mRNA levels were significantly higher in patients treated by RT than in untreated controls (Fig. 2a,b). The median interval between RT and collection of the blood sample was about 10 months.

**Table 1:** Characteristics of breast cancer patients.

Characteristics	Patients, *n* (%)
Number of patients	183 (100)
Median age, years	48
Range	24–84
**Sex**	
Male	10 (5.5)
Female	173 (94.5)
Radiotherapy*	
Yes	162 (88.5)
No	21 (11.5)
**Chemotherapy**	
Yes	118 (64.5)
No	62 (33.9)
NA	3 (1.6)
**Hormone therapy**	
Yes	114 (62.3)
No	69 (37.7)
**Surgery**	
Yes	180 (98.4)
No	2 (1.1)
NA	1 (0.5)
**Stage**	
I	19 (10.4)
II	79 (43.1)
III	58 (31.7)
NA	27 (14.8)
**Recurrence**	
Yes	16 (8.7)
No	167 (91.3)
**Metastases**	
Yes	14 (7.7)
No	169 (92.3)
**Death**	
Yes	6 (3.3)
No	177 (96.7)

Of the 183 BC patients, 180 underwent surgery, 162 were treated by radiotherapy (RT) (including 113 patients receiving both CT and RT, 46 treated by RT alone, and 3 for whom the presence or absence of CT was unknown), 118 received chemotherapy (CT) (including 113 receiving both CT and RT and five patients treated by CT alone), 114 patients received hormone therapy (including 28 treated by RT, 2 treated by CT, 77 treated by CT and RT, and 6 receiving only hormone therapy and have not been included in the 4 groups of BC patients presented in Fig. S3b). Ten patients received no treatment.

*For 22 patients, the date of RT was not available.

In the matched groups of 56 individuals for whom both serum and RNA samples were available, very few patients had undergone only radiotherapy, without associated CT or HT. As we had serum samples from all the individuals of the two initial patient and control cohorts, we analyzed the potential role of radiotherapy in increasing CDA activity in breast cancer patients further by rematching subjects from the two initial cohorts on the basis of age and sex. Each group contained 75 individuals, and we analyzed the CDA activity of all the serum samples from these subjects (the analyses described below were performed on these two matched patient and control groups of 75 individuals each for the purposes of comparison, or on the 183 BC patients from the initial cohort if no comparison with controls was required) (Supplementary Table 2). We compared CDA activity in serum samples from BC patients treated by RT with or without HT (*n* = 13), or treated by RT + CT with or without HT (*n* = 50), with that in serum samples from healthy donors (*n* = 75). We confirmed the increase in CDA activity in serum samples from BC patients treated by RT with or without CT (Fig. 2c), regardless of the time interval between radiotherapy and blood sample collection (Supplementary Fig. 3). Indeed, CDA activity in patient serum did not present any significant variation over time in the matched group and in the initial cohort of RT-treated BC patients (Supplementary Fig. 3a, b, respectively). These results revealed that CT had no impact on the increase in CDA activity in RT-treated BC patients, and suggested that the increase in CDA activity observed in serum samples from BC patients might be due to RT. However, we were unable to exclude an effect of HT, or of the breast cancer itself.

### CDA activity in serum samples from RT-treated BC patients is reduced by anti-estrogen hormone therapy

**Figure 3:**
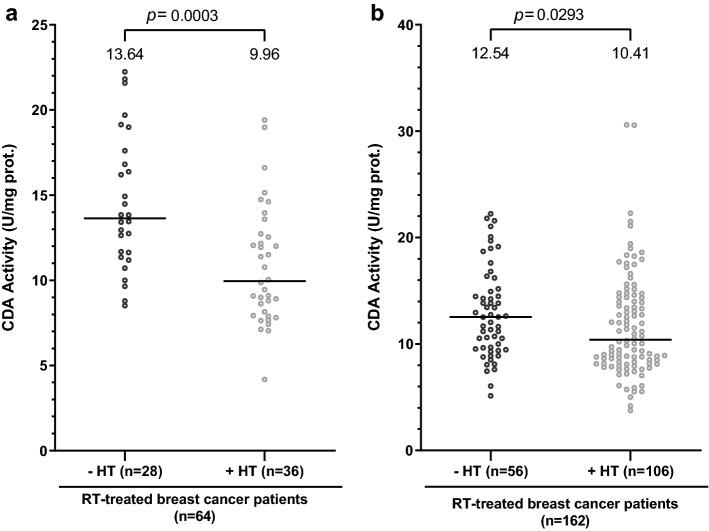
CDA activity in the serum of RT-treated BC patients is significantly reduced by anti-estrogen hormone therapy. (**a**) Scatter plot representation of CDA activity in enzymatic units/mg of protein in matched RT-treated BC patients not treated with HT (in black) and in RT-treated BC patients treated with HT (in gray). (**b**) Scatter plot representation of CDA activity in enzymatic units/mg of protein in RT-treated BC patients from the initial cohort not treated with HT (in black) and in RT-treated BC patients treated with HT (in gray). For each panel, medians are represented by black horizontal lines and are indicated above each distribution. *n* is the number of individuals. Distributions were compared in Mann–Whitney tests (see “Materials and methods” section). Values of *p* < 0.05 were considered statistically significant.

Patients in the matched group treated by RT, with or without CT, were separated into two groups: those treated with HT (*n* = 36), and those not treated with HT (*n* = 28). We compared CDA activity in the serum samples of the patients of these two groups. Activity levels were significantly lower in the serum samples of patients treated with HT (Fig. 3a). We performed the same analysis on the patients of the initial cohort: RT-treated BC patients (*n* = 162) were separated into two groups, with (*n* = 106) and without (*n* = 56) HT. CDA activity levels were significantly lower in the serum samples of BC patients treated with HT (Fig. 3b). Thus, HT reduces CDA activity in the serum of BC patients and cannot be responsible for the increase in CDA activity in the serum samples of RT-treated BC patients.

### Could the increase in CDA activity in the serum of BC patients be due to the breast cancer itself?

It would have been extremely interesting to analyze CDA activity in the serum samples of patients who had received no treatment. However, we had too few BC patients who had undergone only surgery, without RT, CT or HT (10 of 183 in the initial cohort, only six of whom were included in the group of 75 matched patients) for such an analysis. However, despite the impossibility of performing a reliable analysis on so few “untreated” BC patients, we decided to evaluate the median value for CDA activity in the serum samples of these patients. Median CDA activity was 13.20 U/mg protein for the six patients in the matched group, similar to that of RT-treated BC patients (12.00 U/mg protein), but significantly different from that of untreated healthy controls (8.49 U/mg protein) (Supplementary Fig. 4). These results leave open the possibility that the increase in CDA activity observed in the serum samples of BC patients is due to the disease rather than RT.

## Discussion

About 5–10% of breast cancers are of genetic origin and correspond to familial forms. Known high-risk genes, such as *BRCA1, BRCA2, TP53, STK11, CDH1* and *PTEN,* account for less than 20% of the familial forms of breast cancer; most of the genes for which variants or mutations confer a predisposition to cancer development therefore remain unknown^16^.

We recently identified CDA as a new player in the maintenance of genome stability^6–8^ and reported that CDA expression was lost or significantly reduced in about 60% of cancers affecting the general population^17^. These results raised the possibility of a role for constitutive CDA deficiency in some familial forms of breast cancer.

In this study, we analyzed constitutive CDA activity and mRNA levels in blood samples from patients with BC for whom a genetic predisposition was suspected, and healthy volunteers as controls. We detected no CDA deficiency, in terms of either protein activity or mRNA levels, in samples from patients relative to those from controls, and were therefore unable to confirm our initial hypothesis of a relationship between CDA deficiency and cancer predisposition.

Conversely, we found that both CDA activity and CDA mRNA levels were significantly higher in serum samples from BC patients than in those from controls. We also found that CDA activity and mRNA levels were significantly higher in serum samples from RT-treated BC patients than in those of untreated healthy controls. Moreover, our results show that CT has no impact on the increase in CDA activity in RT-treated BC patients. However, given the high levels of CDA expression in the liver and intestines^18,19^, it is conceivable that chemotherapy affects the cells of these organs, leading to an increase in CDA activity, at least during the first few days after CT administration. Unfortunately, we were unable to investigate this possibility, because no blood samples were collected from these patients in the days immediately following CT session.

The treatment of RT-treated BC patients with anti-estrogen hormone therapy was associated with a significant decrease in CDA activity. No relationship between CDA activity and anti-estrogen hormone therapy has ever been reported, highlighting the need for studies to determine how anti-estrogen hormone therapy decreases serum CDA activity.

Finally, we present preliminary data, based on the analysis of CDA activity in the serum of a very small number of BC patients who underwent surgery but not RT, CT or HT, raising the possibility that the increase in CDA activity may be due to the breast cancer itself, rather than RT. We currently have no evidence to suggest that an increase in CDA activity could serve as a prognostic marker of disease progression.

Several studies have suggested that neutrophils are associated with tumor progression, through either an antitumoral or protumoral effect, depending on the tumor microenvironment^20–23^, but we found no correlation between the increase in CDA activity in serum samples and neutrophil counts. These data raise questions about the origin of the increase in CDA activity in the serum of BC patients, which does not appear to be due to neutrophil breakdown or leakage.

Finally, our results also raise the question of the potential practical value of small differences in levels of CDA in BC patients, relative to controls, for diagnosis and prognosis in routine clinical practice. The differences in CDA levels between controls and patients were statistically significant, but the values in the two groups overlapped and were so widely dispersed that it would be difficult to establish threshold values to distinguish between the two groups. However, variations in CDA levels within a given individual may have major clinical implications. Indeed, if the increase in CDA activity and mRNA levels in the blood samples of BC patients is due to the disease itself, then CDA levels in blood could be followed as a marker during routine blood tests in the general population, in a similar manner to the monitoring of prostate-specific antigen (PSA) for prostate cancer^24^: an increase in CDA activity or mRNA levels in the blood over time could be used as a warning signal for the prescription of a mammogram. If the increase in CDA levels is due to RT, then a prospective study would be required to determine whether this increase is of prognostic value for predicting the response to RT. If this is the case, a blood test for CDA before and after RT could improve the anticipation of a risk of relapse, for example. Finally, the decrease in CDA activity in response to hormone therapy may also be of prognostic value for predicting the risk of relapse. Again, a prospective study with a blood test for CDA before and after HT would be required to clarify this point.

In conclusion, in light of our unexpected results, it would be of interest to expand the number of different categories of patients to investigate the significance of the increase in CDA activity and mRNA levels in the blood samples of BC patients over the course of the disease. However, even though we cannot yet determine whether an increase in CDA activity/expression in the blood of patients with breast cancer is of clinical/therapeutic interest for patients, we believe that our results are highly original and open up interesting perspectives for new functional studies.

Moreover, a prospective study conducted as part of a clinical trial would make it possible to determine whether the increase in CDA activity and mRNA levels in the blood of BC patients after treatment is of prognostic value for predicting the course of the disease or the response to treatment, and for determining whether the decrease in CDA activity in response to hormone therapy can be considered an additional benefit of this treatment of potential prognostic value for predicting the risk of relapse. Finally, if the increase in CDA activity in the blood is due to the disease itself, then CDA assays could become a routine test in the general population for the early detection of breast cancer, just as the PSA assay is used for prostate cancer.

## Subjects and methods

### Study subjects

#### Breast cancer patient cohort

In total, 183 consecutive cases of breast cancer patients who had been offered clinical testing for *BRCA1/2* mutations due to the young age at which they were diagnosed and/or family history, participated in the study between June 2010 and February 2011. Clinical follow-up data were available for patients until 2015. Consent for participation in the CDA study was obtained from all the patients during genetic counseling consultations. Blood samples were collected at the same time as samples for the *BRCA1/2* study. Some clinical and biological data were collected, including the date and type of treatment (chemotherapy, radiotherapy, hormone therapy) (Table 1). This cohort consisted of 173 women (94.5%) and 10 men (5.5%) aged from 24 to 84 years, with a median age of 48 years.

#### Healthy donor cohort

We enrolled 196 healthy volunteers in this study as controls, in collaboration with the “Etablissement Français du Sang”, Paris. Written informed consent for research studies was obtained from all participants. This cohort consisted of 102 women (52%) and 94 men (48%), aged from 19 to 70 years, with a median age of 39 years.

All methods were carried out in accordance with relevant guidelines and regulations.

The study protocol was approved by the Institutional Review Board of Institut Curie and was conducted according to the principles of the Declaration of Helsinki.

### Determination of CDA enzymatic activity in serum

Blood samples were collected, left at room temperature for about 30 min, and then centrifuged at 1250 g for 20 min at 4 °C to remove the clot. The resulting serum samples were collected, aliquoted and stored at − 80 °C until analysis. CDA activity was determined as described by Mercier et al.^25^. In brief, the levels of ammonium produced by the conversion of cytidine into uridine by the CDA present in serum were measured by spectrophotometry. Activity is expressed in enzymatic units per mg of protein.

### RNA isolation from blood

Blood was collected in the presence of EDTA. Within an hour of sample collection, erythrocytes were lysed by adding 7.5 mL of EL Buffer (Qiagen) to 1.5 mL of total blood according to the buffer manufacturer’s instructions. Total RNA was isolated from leukocytes with the total RNA isolation mini kit from Macherey–Nagel, in accordance with the manufacturer’s instructions. Nucleic acids were quantified with a Nanodrop spectrophotometer ND-1000 (Thermo Fisher Scientific). RNA quality was checked by electrophoresis in an agarose gel.

### Quantitative RT-PCR

CDA mRNA levels were determined by quantitative RT-PCR. The conditions for cDNA synthesis and RT-qPCR have been described elsewhere^26^. Each sample was normalized on the basis of its *TBP* mRNA content. Results, expressed as N-fold differences in *CDA* gene expression relative to expression of the *TBP* gene, were determined as N = 2^ΔCtsample^, where the ΔCt value of the sample was determined by subtracting the Ct value of the *CDA* gene from the Ct value of the *TBP* gene. The N values of the samples were subsequently normalized such that the median N value for the healthy donor cohort was 1. The nucleotide sequences of the primers used were as follows: *TBP*-U (5′-TGC ACA GGA GCC AAG AGT GAA-3′) and *TBP*-L (5′-CAC ATC ACA GCT CCC CAC CA-3′) for the *TBP* gene, and *CDA*-U (5′-GCA ATT GCT ATC GCC AGT GAC AT-3′) and *CDA*-L (5′-CCA GTT GGT GCC AAA CTC TCT CAT-3′) for the *CDA* gene.

### Statistical analysis

Comparisons between healthy donors and patient subgroups were performed with the two-sided Mann–Whitney tests or Kruskal–Wallis test for more than two groups. Comparisons between matched patients and controls were performed with the Wilcoxon matched-pairs signed-rank test. Matching of patients and healthy donor was realized using the function Match of the R package “Matching”. The two groups were matched on sex and age, without replacement. The distance considered as acceptable for the matching on age was set at 0.35 standard deviations^27^. The potential relationship between CDA activity and neutrophil counts was assessed in a Pearson’s correlation test. All the statistical tests were performed in GraphPad Prism. We considered *p* values < 0.05 to be significant.

## Supplementary Information

Supplementary Information 1.

Supplementary Information 2.

Supplementary Information 3.
