# Author Correction: Cytidine deaminase activity increases in the blood of breast cancer patients

**DOI:** 10.1038/s41598-022-23982-4

**Published:** 2022-12-05

**Authors:** Géraldine Buhagiar‑Labarchède, Rosine Onclercq‑Delic, Sophie Vacher, Frédérique Berger, Ivan Bièche, Dominique Stoppa‑Lyonnet, Mounira Amor‑Guéret

**Affiliations:** 1 Institut Curie, PSL Research University, UMR 3348, 91405 Orsay, France; 2 CNRS UMR 3348, Centre Universitaire, Bat 110, 91405 Orsay, France; 3 Université Paris-Saclay, Centre Universitaire, Bat 110, UMR 3348, 91405 Orsay, France; 4 Department of Genetics, Institut Curie, Paris, France; 5 Department of Biostatistics, Institut Curie, Paris, France; 6 Université Paris Cité, Paris, France; 7 INSERM U830, Institut Curie, Paris, France

Correction to: *Scientific Reports* https://doi.org/10.1038/s41598-022-18462-8, published online 18 August 2022

The Supplementary Information 1 file published with this Article contained a wrong Supplementary Figure 1.

This error has now been corrected in the Supplementary Information 1 file that accompanies the original Article.

## Supplementary Information

Supplementary Information.

