# Supplementary material for: Cytidine deaminase activity increases in the blood of breast cancer patients
Source: Sci Rep. 2022 Aug 18;12:14062. doi: 10.1038/s41598-022-18462-8 (PMC9388666; doi:10.1038/s41598-022-18462-8)
Supplement: Supplementary file 1 — Supplementary Information 1. [file 41598_2022_18462_MOESM1_ESM.pdf]

Supplementary Information

Cytidine deaminase activity increases in the blood of breast cancer patients

Géraldine Buhagiar-Labarchède[1,2,3], Rosine Onclercq-Delic[1,2,3], Sophie Vacher[4], Frédérique Berger[5], Ivan Bièche[4,6], Dominique Stoppa-Lyonnet[4,6,7], Mounira Amor-Guéret[1,2,3,8*]

[1]Institut Curie, PSL Research University, UMR 3348, 91405, Orsay, France

[2]CNRS UMR 3348, Centre Universitaire, 91405, Orsay, France

[3]Université Paris-Saclay, Centre Universitaire, UMR 3348, 91405, Orsay, France

[4]Department of Genetics, Institut Curie, Paris, France

[5]Department of Biostatistics, Institut Curie, Paris, France.

[6]Université Paris Cité, France

[7]INSERM U830, Institut Curie, Paris, France

[8]Lead contact

[*]Correspondence: mounira.amor@curie.fr

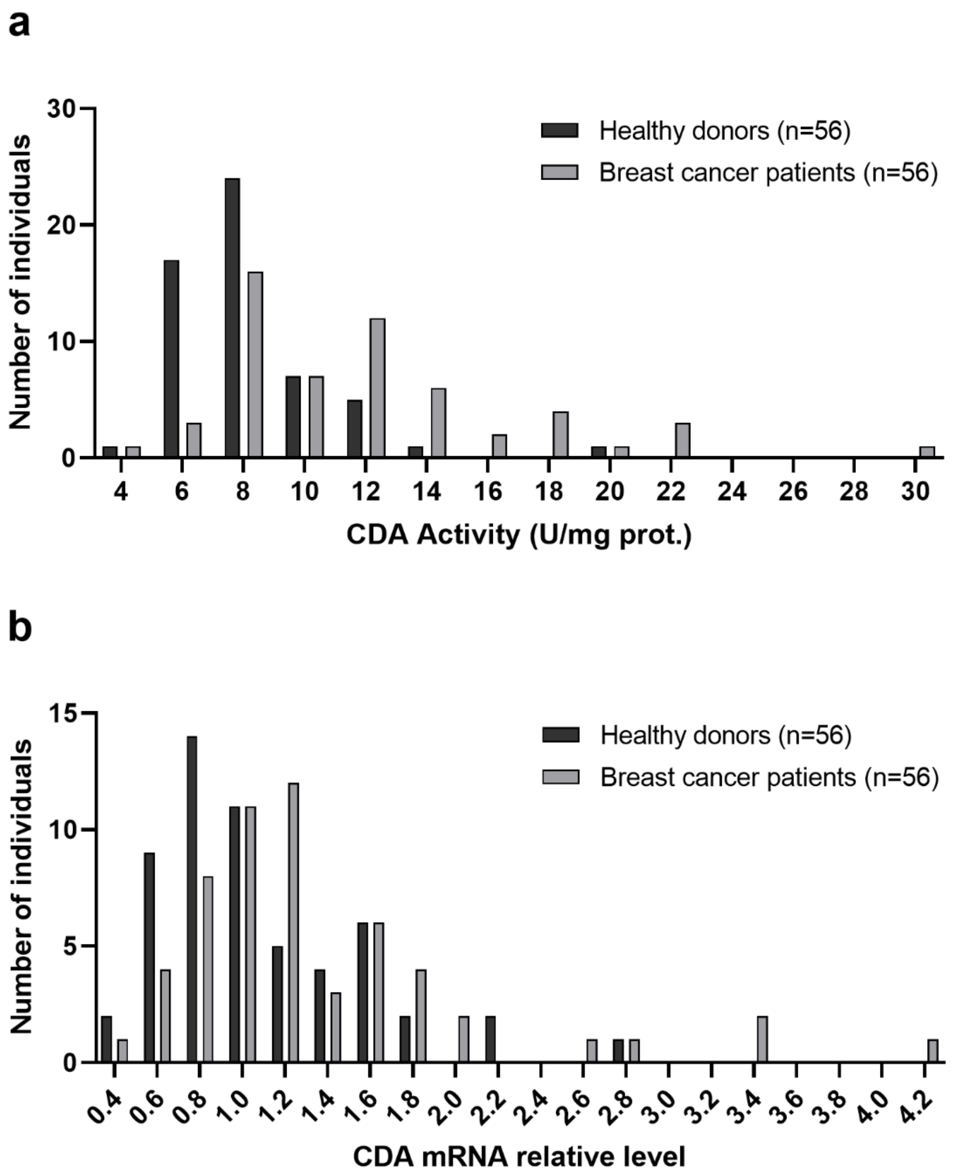

Supplementary Figure 1. The lowest values of CDA activity or expression were found in healthy donors, and the highest values were found in BC patients. **(a)** Distribution of CDA activity in enzymatic units/mg of protein in matched healthy donors (in black) and BC patients (in gray). **(b)** Distribution of relative levels of CDA mRNA in matched healthy donors (in black) and BC patients (in gray).

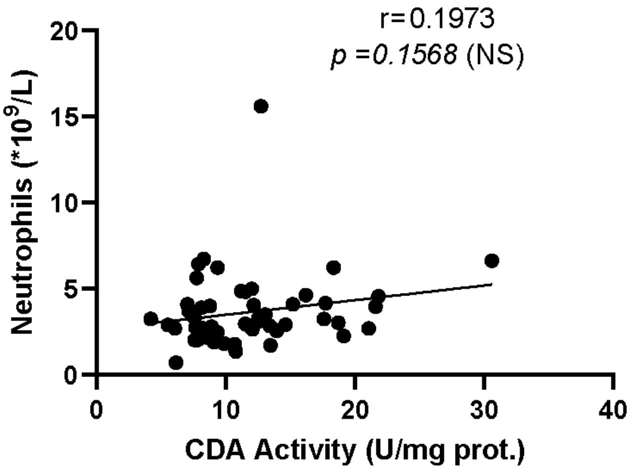

Supplementary Figure 2. Scatter plot of the correlation between CDA activity in enzymatic units/mg of protein and the number of neutrophils in the 56 matched BC patients. Significance was assessed by calculating Pearson's coefficient of correlation. NS (non-significant) indicates a *p*-value >0.05.

a

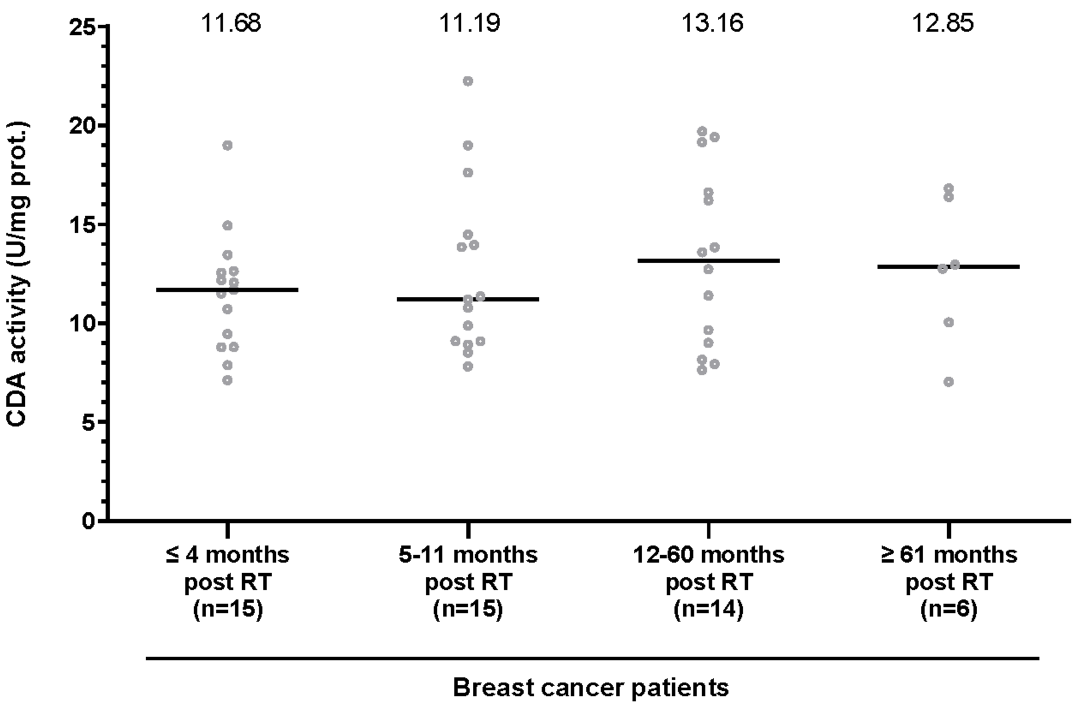
11.68
11.19
13.16
12.85
CDA activity (U/mg prot.)
25
20
15
10
5
0
≤ 4 months post RT (n=15)
5-11 months post RT (n=15)
12-60 months post RT (n=14)
≥ 61 months post RT (n=6)
Breast cancer patients

b

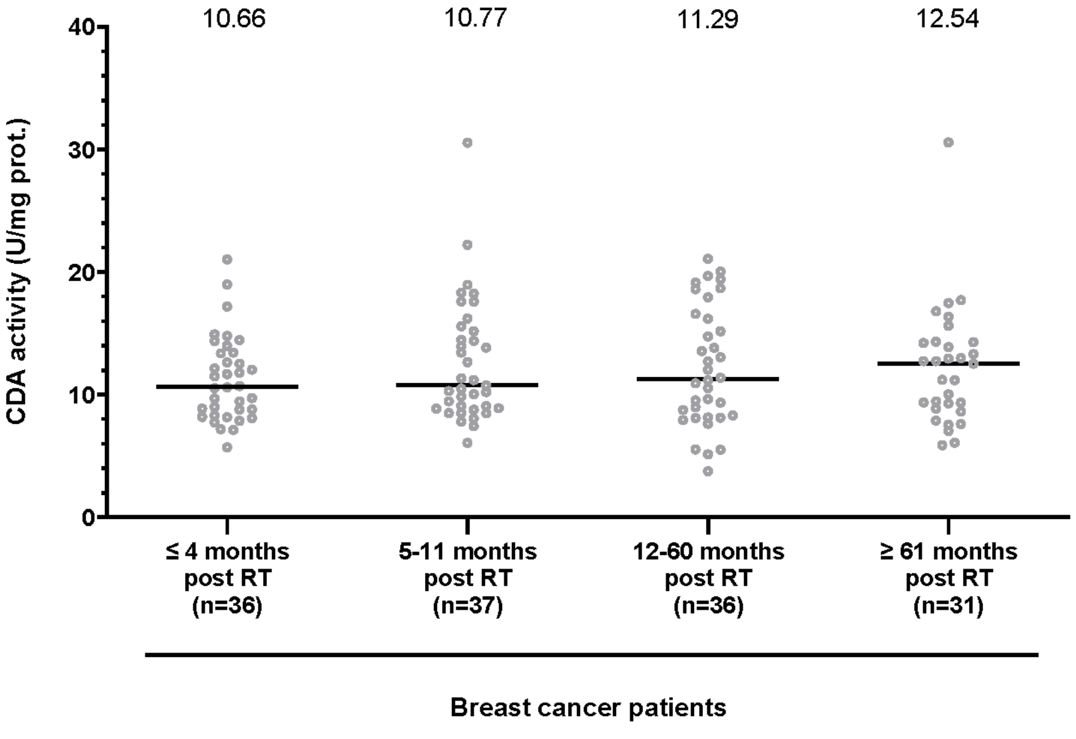
10.66
10.77
11.29
12.54
CDA activity (U/mg prot.)
40
30
20
10
0
≤ 4 months post RT (n=36)
5-11 months post RT (n=37)
12-60 months post RT (n=36)
≥ 61 months post RT (n=31)
Breast cancer patients

Supplementary Figure 3. CDA activity in BC patients remained stable over time after RT. Scatter plot representation of CDA activity in enzymatic units/mg of protein in the matched group **(a)** or the initial cohort **(b)** of BC patients according to the time interval between RT and blood sample collection (14 and 22 patients, respectively, for whom the date of RT was unknown, were not included in these analyses). Medians are represented by black horizontal lines and are indicated above each distribution. n is the number of individuals. Distributions were compared in Kruskal-Wallis tests; no significant differences between medians were detected.

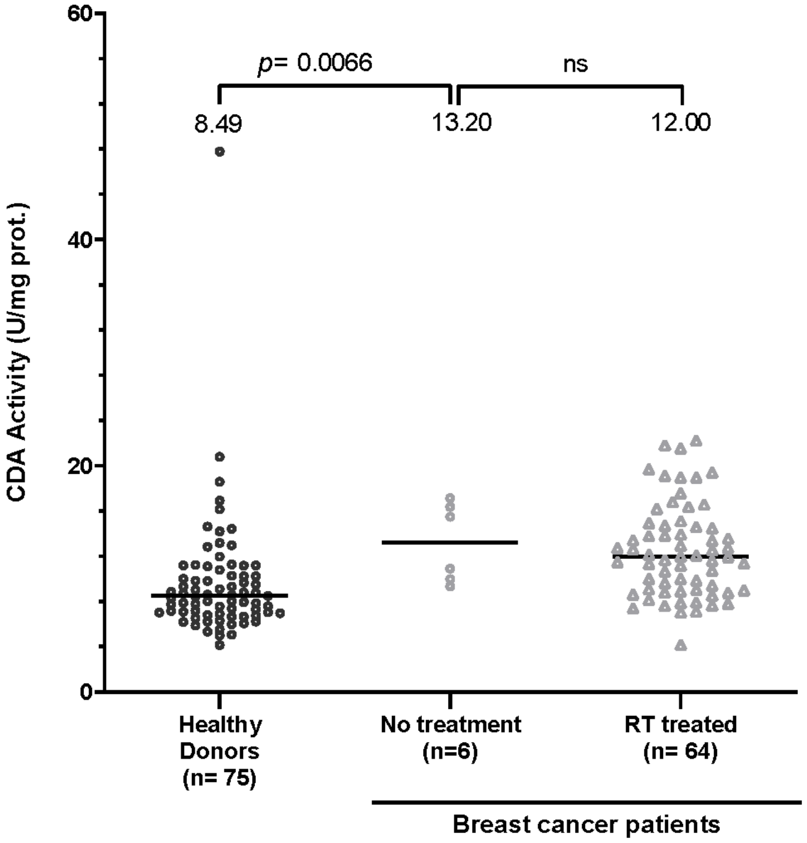

Supplementary Figure 4. CDA activity levels are significantly higher in the serum of "untreated" BC patients (patients who had undergone surgery only) than in those from untreated healthy donors. Scatter plot representation of CDA activity in enzymatic units/mg of protein

from the 75 matched group: in 75 healthy donors (circle in black) versus 70 BC patients, 6 “untreated” patients (circle in gray) and 64 only RT-treated BC patients (triangles in gray).

Supplementary Table 1. Characteristics of the 56 matched breast cancer patients. ***Provided as a separate file (.xlsx)***

Supplementary Table 2. Characteristics of the 75 matched breast cancer patients. ***Provided as a separate file (.xlsx)***
